# SUrgical versus PERcutaneous Bypass: SUPERB-trial; Heparin-bonded endoluminal versus surgical femoro-popliteal bypass: study protocol for a randomized controlled trial

**DOI:** 10.1186/1745-6215-12-178

**Published:** 2011-07-18

**Authors:** Mare MA Lensvelt, Suzanne Holewijn, Wilbert M Fritschy, Otmar RM Wikkeling, Laurens A van Walraven, Bas M Wallis de Vries, Clark J Zeebregts, Michel MPJ Reijnen

**Affiliations:** 1 Rijnstate Hospital, Department of Surgery, Arnhem, The Netherlands; 2 Isala Klinieken, Department of Surgery, Zwolle, The Netherlands; 3 Nij Smellinghe Hospital, Department of Surgery, Drachten, The Netherlands; 4 Antonius Hospital, Department of Surgery, Sneek, The Netherlands; 5 University Medical Center Groningen, Department of Surgery, Division of Vascular Surgery, Groningen, The Netherlands

**Keywords:** superficial femoral artery, femoro-popliteal bypass, endoluminal, stentgraft, peripheral arterial occlusive disease

## Abstract

**Background:**

Endovascular treatment options for the superficial femoral artery are evolving rapidly. For long lesions, the venous femoropopliteal bypass considered to be superior above the prosthetic bypass. An endoluminal bypass, however, may provide equal patency rates compared to the prosthetic above knee bypass. The introduction of heparin-bonded endografts may further improve patency rates. The SUrgical versus PERcutaneous Bypass (SuperB) study is designed to assess whether a heparin-bonded endoluminal bypass provides equal patency rates compared to the venous bypass and to prove that it is associated with improved quality of life, related to a decreased complication rate, or not.

**Methods/design:**

Two-hundred-twenty-two patients with peripheral arterial occlusive disease, category 3-6 according to Rutherford, will be randomized in two treatment arms; 1. the surgical femoro-popliteal bypass, venous whenever possible, and 2. the heparin-bonded endoluminal bypass. The power analysis was based on a non-inferiority principle, with an effect size of 90% and 10% margins (alpha 5%, power 80%). Patients will be recruited from 5 teaching hospitals in the Netherlands during a 2-year period. The primary endpoint is primary patency and quality of life evaluated by the RAND-36 questionnaire and the Walking Impairment Questionnaire. Secondary endpoints include secondary patency, freedom-from-TLR and complications.

**Discussion:**

The SuperB trial is a multicentre randomized controlled trial designed to show non-inferiority in patency rates of the heparin-bonded endograft compared to the surgical bypass for treatment of long SFA lesions, and to prove a better quality of life using the heparin bonded-endograft compared to surgically treatment, related to a reduction in complications.

**Trial Registration**

Clinicaltrials: NCT01220245

## Background

In the treatment of long lesions of the superficial femoral artery (SFA) the surgical venous bypass is considered the gold standard [1]. Bypass surgery, however, is associated with complications and a prolonged hospital length of stay. Endovascular techniques have advanced and provided new treatment options for peripheral vascular disease, but may also induce complications as shown in the Basil trial [2]. Last few years evidence has been accumulating that the treatment of long lesions with (covered) stents may provide acceptable short- and midterm primary patency rates, as summarized in several recent reviews [3-7].

Randomized trials have shown that 1-year patency rates of self-expanding nitinol metal stents vary between 70-90% as recently summarized by Lin et al [5]. Despite improvements in stent design and introducer sizes, one of the major issues limiting patency is in-stent re-stenosis. It was recently demonstrated that with the use of covered stents, re-stenosis is reduced to edge stenosis only [8]. The efficacy of an ePTFE-covered nitinol stent (Viabahn, W.L. Gore, Flagstaff, AZ, USA) in treating chronic SFA long lesions, of over 8 cm length, is currently being compared to bare nitinol stents in a multicentre randomized controlled trial (VIBRANT trial). The study hypothesis is that the use of ePTFE-covered nitinol stents will result in greater mid-term (24 months) and long-term (36 months) patency. Although interim analysis has shown no significant differences at one-year, final results have to be awaited. Recently, the 4-year's results of a randomized trial have been published comparing an ePTFE-covered nitinol stent with the above-knee ePTFE femoropopliteal bypass [9]. The 1-year and 2-year patency rates were 73% and 74%, and 63% and 64%, respectively [10,11] At 4-years of follow-up, the primary patency rates were 59% and 58%, respectively and limb salvage rates were also comparable. When compared to a prosthetic bypass, however the venous bypass has better patency rates, with 1-year and 4-year primary patency rates of 87% and 70%, respectively. There are patient groups with long lesions of the SFA, including patients with severe co-morbidities, who might benefit from a less invasive treatment strategy. The risk of wound complications, limb edema, loss of the great saphenous vein, and cardiac complications may have to be taken into account when deciding to treat surgically or endovascularly.

Heparin-bonded prosthetic bypass grafts have shown improved patency rates in animal models and non-randomized clinical trials [12-14]. Recently, the heparin-bonding technology has been integrated within the Viabahn stentgraft. Using this technique results may further improve to the level of the current gold standard; the venous femoropopliteal bypass. Advantages of the endoluminal technique would be related to its minimal invasive character: less pain, earlier recovery and less early complications. To date, no studies have been performed to compare the use of heparin-bonded stent grafts for the treatment of long lesions of the SFA. The current study has been designed to compare the use of heparin-bonded stent grafts for the treatment of long lesions of the SFA to the venous surgical femoropopliteal bypass in a multicentre randomized controlled trial.

## Methods and design

### Study design

The design of the study is a multicentre prospective randomized controlled trial comparing the patency of the heparin-bonded endograft to the venous surgical bypass. Endpoints are primary patency after 1, 2 and 5 years, complications and quality of life.

### Study objectives

The aim of the study is to demonstrate that the heparin-bonded endograft provides equal patency rates compared to the venous surgical femoro-popliteal bypass. In addition we hypothesize that patients receiving a heparin-bonded endograft show better quality of life at 30-days compared to patients who were surgically treated.

### Sample size calculation

The assumption has been made that the heparin-bonded endoluminal bypass will have a similar cumulative primary patency at one year compared to the venous bypass. For a non-inferiority trial with an effect size of 90% and a margin of 10%, 111 patients per group are needed (alpha 5%, power 80%). The effect size of 90% refers to an estimated patency rate at one year in the surgical control arm.

The assumption has been made that the heparin-bonded endoluminal bypass will have an increase in QOL, as measured by a 10-point increase in the SF-36 score, at 30 days follow-up. With a standard deviation of 20, 63 patients per group are needed (alpha 5%, power 80%).

### Setting

Patients will be recruited from the following centers: Rijnstate Hospital, Arnhem; Isala Klinieken, Zwolle; Nij Smellinghe Hospital, Drachten; Antonius Hospital, Sneek; University Medical Center Groningen, Groningen, The Netherlands. In each of the participating centers, each surgeon performing the endovascular procedure must have placed at least ten endoluminal bypasses prior to treating patients who participate in the SuperB trial to prevent a learning curve bias.

The total study duration will be 7 years; the recruitment period will take 2 years and thereafter patients will be evaluated yearly until 5 years post-procedure.

### Primary endpoints

The primary endpoints of the study are the primary patency at 1-year follow-up. In addition the quality of life, using the RAND SF36 Questionnaire will be evaluated as a primary endpoint.

### Secondary endpoints

1. Secondary patency

2. Complications

3. Clinical improvement

4. Surgical and endovascular re-interventions

5. Target lesion revascularization

Additionally an exploratory, thus hypothesis generating, subgroup-analysis will be performed.

- Patients with disabling intermittent claudication (Rutherford 3) will be analyzed separately using pain-free and maximal walking distance and the Walking Impairment Questionnaire as additional endpoints.

- Patients with ischemic rest pain and necrosis (Rutherford 4-6) will be analyzed using major amputations as an endpoint.

### Ethical considerations

A patient who meets the entry criteria is fully informed about the trial and provided with a patient information and consent form. Patients willing to participate in the study are included after signing the informed consent form. This study is conducted in accordance with the principles of the Declaration of Helsinki and Good Clinical Practice guidelines. The study is approved by the Medical Ethics committee of Nijmegen (CMO 2010-089) and the local institutional board of each participating center.

### Safety and quality control

#### Data Safety Monitoring Board

The Data Safety Monitoring Board (DSMB) will review safety and makes recommendations regarding the conduct of the study to the steering committee and to the accredited Medical Ethical Board (METC) that approved the study protocol. An interim safety analysis will be performed at 1-year after initiation of the trail. This analysis will include at least 40 patients.

#### Adverse and severe adverse events

Adverse events (AE) are defined as any undesirable experience occurring to a participant during the study, whether or not considered related to the investigational device. This definition includes events occurring during hospital stay up to 30 days of follow-up. Underlying disease that was present at the time of enrollment is not reported as an AE, but any increase in the severity of the underlying disease will be reported as an AE. All AEs will be monitored from the time of enrolment through the 30-day follow-up visit. AEs will be recorded on the case record forms (CRFs). A description of the event, including the start date, end date, action taken, and the outcome will be provided.

A severe adverse event (SAE) is any event leading to death, major amputation or definitive graft failure.

Data on AEs will be reported to the DSMB and to the accredited METC via "Toetsingonline" on the website of the Central Committee on Research involving Human Subjects (CCMO, ccmo.nl).

### Inclusion criteria

· Patients over 18 years of age

· Informed consent

· De novo stenosis, re-stenosis (Peak Systolic Velocity (PSV) ratio > 2.5) or occlusion of the native SFA, all > 10 cm in length

· Popliteal artery is patent at the upper margin of the patella to the trifurcation

· Diameter of the native SFA and popliteal arteries are 5.0 to 7.5 mm

· Rutherford category 3-6

· Indication for surgical bypass

· Distal runoff at least 1 crural vessel without significant stenosis

· Resting ankle-brachial index (ABI) < 0.8 in the study limb prior to procedure

### Exclusion criteria

· Patient unsuitable for administration of contrast agent

· Pregnancy

· Dementia or altered mental status that would prohibit giving conscious informed consent

· Need for adjunctive major surgical or vascular procedures within 1 month

· Untreated flow-limiting aorto-iliac occlusive disease

· Unsuccessful ipsilateral percutaneous vascular procedure to treat inflow disease just prior to enrollment

· Previous ipsilateral bypass surgery or stent-placement

· Femoral or popliteal aneurysm of target vessel

· Non-atherosclerotic disease resulting in occlusion (e.g. embolism, Buerger's disease, vasculitis)

· Severe medical co morbidities (untreated coronary artery disease/congestive heart failure, severe chronic obstructive pulmonary disease, metastatic malignancies, dementia, etc.) or other medical condition that would preclude compliance with the study protocol

· Major distal amputation (above the trans metatarsal) in the study limb

· Any previously known coagulation disorder, including hypercoagulability

· Contraindication to anticoagulation or antiplatelet therapy

· Known allergies to stent or stent-graft components

· History of prior life-threatening reaction to contrast agent

· Patients with known hypersensitivity to heparin, including those patients who have had a previous incidence of heparin-induced thrombocytopenia (HIT) type II

· Planned surgical procedure or major amputation to occur after enrollment of the patient

### Recruitment

Patients with symptomatic peripheral arterial disease of the superficial femoral artery with a Rutherford category 3-6 [15] may be included in the study and will be recruited among the 5 participating centers.

### Randomization

Randomization will be performed during the out patient department visit in which the patient is included. The including physician will call the telephone number provided by the principle investigator. The person answering the phone will draw an envelope from a box of plain white envelopes containing the randomization choice. The envelopes are divided in batches of 20 and randomization is stratified by center.

### Imaging

Ultrasound imaging screens all patients included in the study. Additionally, computed tomography angiography (CTA) or magnetic resonance angiography (MRA) will be performed. These imaging studies will be performed according to the local protocol of the participating centers. The lesions in the SFA will be categorized according to the Trans Atlantic Intersociety Consensus (TASC)-II criteria. Type B, C, and D lesions may be included.

## Treatment details

### Endovascular technique

Antibiotic prophylaxis is administered. Preferably a percutaneous technique is used, but in case of a flush occlusion or a diseased common femoral artery, an open approach is allowed. The SFA may be approached in a contralateral retrograde or an ipsilateral antegrade fashion. When there is a concomitant lesion in the common or profunda femoral artery an endarterectomy may be performed followed by the endoluminal bypass. Heparin (5000 I.U.) is administered. The diseased segment of the SFA is passed, either endoluminal or sub-intimal and a re-entry is created distally. The segment is pre-dilated with a regular angioplasty balloon and the endografts are positioned from distal to proximal without or with minimal or no oversizing. The entire diseased segment is covered with the stentgrafts and the stentgrafts are post dilated with an angioplasty balloon with the same size as the stent graft. Control angiography is performed routinely and the access is closed using a closure device, according to local protocols.

The used stent graft is the heparin-bonded Viabahn Endoprothesis (W.L. Gore & associates, Flagstaff, AZ), which is a self-expanding helical nitinol stent covered with a heparin-bonded thin polytetrafluorethylene (ePTFE) tube. The size of the stentgraft should be at least 6 mm.

Post procedurally, all patients will be treated with acetylsalicylic acid 80 mg and clopidogrel 75 mg for the first year unless oral anticoagulation is indicated for other reasons. After 1 year patients may be switched to 1 thrombocyt aggregation inhibitor. All patients receive statin treatment, started before the intervention.

### Open surgical technique

The surgical femoropopliteal bypass is performed according to local protocols. Preferably the greater saphenous vein is used as conduit in all patients. The used vein has a diameter of at least 3.5 mm. Pre-operative vein mapping may be performed, but is not obligatory. When the great saphenous vein is unavailable or unsuitable a prosthetic graft may be used. When a prosthetic conduit is used the participating surgeon will explicit this choice. All patients will be included in the intention-to-treat analysis.

Post procedurally, all patients will be treated with acetylsalicylic acid 80 mg and clopidogrel 75 mg for the first year unless oral anticoagulation is indicated for other reasons. After 1 year patients may be switched to one thrombocyt aggregation inhibitor. All patients receive statin treatment, started before the intervention.

### Follow-up

Follow-up is planned at 1, 3, and 6 months. Afterwards patients will be seen each 6 months until 2 years. From 2 to 5 years patients will be evaluated annually. Duplex ultrasound imaging, ankle-brachial indices, and QOL scores will be measured at all above mentioned time points. All primary and secondary endpoint are registered as defined in the study protocol.

### QOL scores

• RAND-36 is a multidimensional measurement of health. This will also be used in both groups

• WIQ (Walking Impairment Questionnaire) is especially designed for patients with claudication, and will only be used in patients treated for claudication.

These scores are taken prior to the intervention and at defined times afterwards (i.e. 1 day, 1 week, 1 month, etc).

### Data collection

Data will be collected at the recruitment centre by means of case report forms (CRF's). The copies of the CRF forms will be sent to the coordinating center (Rijnstate Hospital) where all data will be entered in the central database and controlled by an independent monitor. The participating centers will be informed about the current status of recruitment and adverse events via a newsletter every 3 months. Additionally, there will be regular contact between the principle investigator and the contact persons from the participating centers.

### Statistical analyses

Data concerning the 1, 2, and 5 year follow-up will be analyzed for both study groups on an intention-to-treat and a per-protocol manner by student t-test (normal distribution) of Mann Whitney U-test (skewed distribution). Corrections will be made for study centre. Additionally, in case of sufficient numbers, the data will be analyzed for different TASC II categories; otherwise the analyses will be additionally adjusted for TASC II categories. Patency rates will be presented as Kaplan Meier curves including censoring.

### Publication of data

Data will be published after a follow-up period of 1, 2, and 5 years, regardless of the outcome of the study under the responsibility of dr. MMPJ Reijnen. Co-authorship will be assigned according to the ' Uniform Requirements for Manuscripts Submitted to Biomedical Journals: Writing and Editing for Biomedical Publication' of the International Committee of Medical Journal Editors.

### Definitions

• Procedural success:

○ Endovascular - Successful vascular access, exact deployment of the device and completion of the endovascular procedure and immediate morphological effect (< 30% residual stenosis)

○ Surgical - Successful access, completion of surgical procedure and clinically assessed immediate improvement

• Primary patency: The absence of occlusion or flow-limiting stenosis (PSV ratio > 2.5) of the treated segment of the artery including 1 cm proximal and distal of the anastomosis, as documented by accepted imaging techniques, particularly arteriography or duplex ultrasonography, or direct observation at operation or postmortem.

• Primary assisted patency: When a secondary endovascular or open procedure is performed to prevent failure, i.e. in a flow reducing stenosis (PSV ratio > 2.5), in a still-patent segment of the stent graft or bypass, including the anastomoses.

• Secondary patency: When a thrombolytic or surgical treatment has been performed for graft or stent graft occlusion in an afterwards patent vessel.

• Target lesion revascularization: Repeat percutaneous or surgical revascularization driven by clinical state in the presence of a flow reducing stenosis or occlusion in the treated segment of the artery including 1 cm proximal and distal of the anastomosis.

• Type B lesion SFA according to TASC II criteria:

○ Multiple lesions (stenosis or occlusions), each ≤ 5 cm

○ Single stenosis or occlusions ≤ 15 cm (not involving infragenuale popliteal segment)

○ Heavily calcified occlusion ≤ 5 cm in length

○ Single popliteal stenosis

• Type C lesion SFA according to TASC II criteria:

○ Multiple stenosis or occlusions totaling > 15 cm with or without heavy calcification

○ Recurrent stenosis or occlusions that need treatment after 2 endovascular interventions

• Type D lesion SFA according to TASC II criteria:

○ Chronic total occlusions of CFA or SFA (> 20 cm, involving the popliteal artery)

○ Chronic total occlusions of popliteal artery and proximal trifurcation arteries

• Minor amputation: Below the ankle amputation, planned or unplanned.

• Major amputation: Above the ankle amputation, planned or unplanned

• Clinical improvement: Improved Rutherford classification compared to baseline.

• Intermediate lesion: Occlusion or stenosis > 10 cm length

• Flow-reducing (re-)stenosis: A stenosis with a PSV ratio of more than 2.5 as measured by duplex, or a > 50% (re-)stenosis on angiography, MRA or CTA.

• Graft failure: Definitive occlusion of the bypass with unsuccessful thrombolytic or surgical treatment

• Re-intervention: Secondary percutaneous or surgical intervention of the bypass.

• Seroma - Non-infected fluid accumulation under the wound

• Hematoma - Accumulation of blood postoperatively in the operated area.

• Re-bleed - Accumulation of blood postoperatively requiring operative treatment.

• Abscess - Accumulation of pus in the operated area.

• Infected wound - Red, swollen, but closed wound, not requiring surgical drainage.

• Open wound - Non infected wound leaking fluid.

• Loss of sensibility - Postoperative clinical loss of sensibility of the skin in the operated leg.

• Femoral nerve damage - Clinical femoral nerve damage.

• Graft infection - Proven graft infection requiring long term use of antibiotics or graft removal.

• Edema - Postoperative persistent edema of the operated leg.

• Serious adverse event (SAE): An adverse event that leads to death or serious deterioration in the health of the subject, defined as death, definite failure of graft/bypass or major amputation in the treated leg

## Discussion

The aim of the present randomized trial is two-fold. First, it aims to compare patency rates of the endoluminal bypass, combined with the heparin-bonded technology, with the current gold standard, the venous femoro-popliteal bypass, the current gold standard. Second, it aims to demonstrate that an endovascular approach is associated with fewer complications and thus an improved quality of life. In order to have sufficient power, the trial was designed using a non-inferiority principle.

Data analysis will be performed in both an intention-to-treat as a per-protocol manner. The first will be performed to compare the endoluminal bypass with common surgical practice, namely that some patients will not have a vein usable for the bypass and that some endoluminal procedures will be converted to open surgery. The per-protocol analysis will give information about the performance of the endoluminal bypass itself.

There are several differences between the SuperB trial and previously published randomized trials [9,16]. The SuperB trial is the first trial using a heparin-bonded endograft and the inclusion criteria are chosen much broader since hybrid procedures, such as a reconstruction of the common femoral artery, are allowed. Moreover, the anti-platelet regime and the use of statins are standardized. The wide inclusion criteria are chosen because they mimic common surgical practice and thereby they reduce the risk on inclusion problems as was the case in the Scandinavian trial [16].

**Figure 1:**
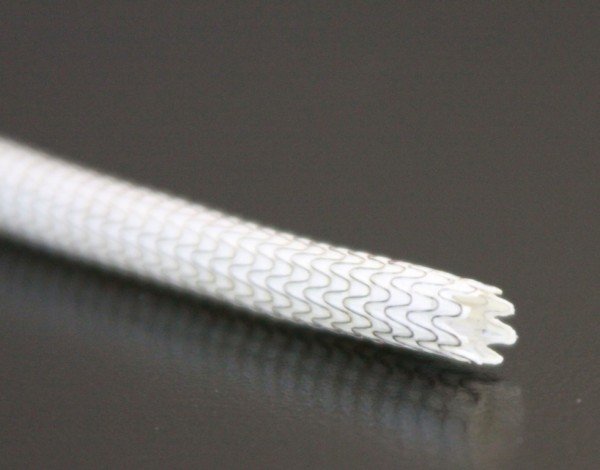
**The Viabahn endograft (W.L. Gore, Flagstaff, AZ, USA) with a contoured proximal edge**.

With the introduction of the heparin-bonded technology in the endograft the design of the stent has also been changed. The proximal edge of the endograft has no longer a straight, but a contoured edge (Figure 1). This adaptation will reduce infolding in case of oversizing, thereby maintaining laminar blood flow and preventing intimal hyperplasia and thus edges stenosis. The expected effect of the new endograft may therefore not only be attributed heparin-bonding technology.

The SuperB trial hypothesizes that the treatment of the long lesion of the SFA with a heparin-bonded endograft will show equal patency rates compared to the surgical bypass, but a better quality of life for treatment with the heparin-bonded endograft is expected, related to a reduced morbidity. Therefore, patients will be asked to fill out the RAND 36 questionnaire before treatment and at each visit during follow-up. Patients with intermittent claudication additionally will be asked to fill out the Walking Impairment Questionnaire at the same visits. Trials on endoluminal bypass including quality of life scores have not been performed, to date.

In conclusion, the SuperB trial is a multicentre randomized controlled trial designed to show equality in patency rates of the heparin-bonded endograft compared to the surgical bypass for treatment of longer lesions of the SFA, but to show better quality of life using the heparin bonded-endograft compared to surgically treatment, related to a reduction in complications.

## List of abbreviations

ABI: Ankle brachial index; AE: Adverse events; CCMO: Central Committee on Research involving Human Subjects; CAD: Coronary artery disease; CFA: Common femoral artery; CHF: Congestive heart failure; COPD: Chronic Obstructive Pulmonary Disease; CT: Computed tomography; CRF: Case record forms; DSMB: Data Safety Monitoring Board; ePTFE: Polytetrafluorethylene; HIT: heparin-induced thrombocytopenia; METC: Medical Ethical Board; MRA: Magnetic resonance angiography; PSV: Peak systolic velocity; SAE: Severe adverse event; SFA: Superficial femoral artery; SF36: 36-Item Short form health survey; WIQ: Walking Impairment Questionnaire.

## Competing interests

The authors declare that they have no competing interests. This is an investigator-sponsored study supported in part by W.L. Gore and Associates.

## Authors' contributions

ML and MR drafted the manuscript.

SH, WF, OW, LvW, VWdV, and CZ participated in the design of the study.

All authors edited the manuscript and read and approved the final manuscript.
